# *Propionibacterium acnes* prosthetic valve endocarditis with abscess formation: a case report

**DOI:** 10.1186/1471-2334-14-105

**Published:** 2014-02-25

**Authors:** Mario Kurz, Beat A Kaufmann, Larry M Baddour, Andreas F Widmer

**Affiliations:** 1 Division of Infectious Diseases and Hospital Epidemiology, University Hospital Basel, Basel, Switzerland; 2 Division of Cardiology, University Hospital Basel, Basel, Switzerland; 3 Division of Infectious Diseases, Mayo Clinic, 200 First Street S.W, Rochester, MN 55905, USA

**Keywords:** Endocarditis, *Propionibacterium acnes*, Biofilm, Rifampin

## Abstract

**Background:**

Endocarditis due to *Propionibacterium acnes* is a rare disease. Scant data on treatment of these infections is available and is based on case reports only. If the disease is complicated by abscess formation, surgical intervention combined with an antibiotic therapy might improve clinical outcome. In some cases, cardiac surgeons are reluctant to perform surgery, since they consider the intervention as high risk. Therefore, a conservative therapy is required, with little, if any evidence to choose the optimal antibiotic. We report the first case of a successfully treated patient with *P. acnes* prosthetic valve endocarditis without surgery.

**Case presentation:**

We report the case of a 29-year-old patient with a prosthetic valve endocarditis and composite graft infection with abscess formation of the left ventricular outflow tract due to *P. acnes*. Since cardiac surgery was considered as high risk, the patient was treated intravenously with ceftriaxone 2 g qd and rifampin 600 mg bid for 7 weeks and was switched to an oral therapy with levofloxacin 500 mg bid and rifampin 600 mg bid for an additional 6 months. Two sets of blood cultures collected six weeks after completion of treatment remained negative. The patient is considered to be cured based on absence of clinical signs and symptoms, normal laboratory parameters, negative radiology scans and negative blood cultures, determined at site visits over two years after completion of treatment.

**Conclusion:**

To our knowledge, this is the first successfully managed patient with *P. acnes* prosthetic valve endocarditis with abscess formation of the left ventricular outflow tract who was treated with antibiotics alone without a surgical intervention. A six month treatment with a rifampin and levofloxacin combination was chosen, based on the excellent activity against stationary-phase and adherent bacteria.

## Background

Endocarditis due to *Propionibacterium acnes* is a rare disease after prosthetic valve replacement [1,2]. Diagnosis is often delayed due to oligosymptomatic manifestation resulting in valvular and perivalvular destruction or abscess formation [3]. Scant data on treatment of these infections is available and is based on case reports only. If the disease is complicated by abscess formation, the combination of surgical intervention and antibiotic therapy might lead to a favourable clinical outcome as demonstrated in several case reports [4]. *P. acnes* has the ability to form biofilm, leading to diminished susceptibility of the organism to most antibiotics. As already shown for staphylococci, rifampin is also active against biofilm forming *P. acnes*[5]. In some cases, cardiac surgeons are reluctant to perform surgery, since they consider the interventions as high risk. Therefore, a conservative therapy is required, with little, if any evidence to choose the optimal antibiotic. We report the first case of a successfully treated patient with *P. acnes* prosthetic valve infection with abscess formation without surgery.

### Case presentation

**Figure 1:**
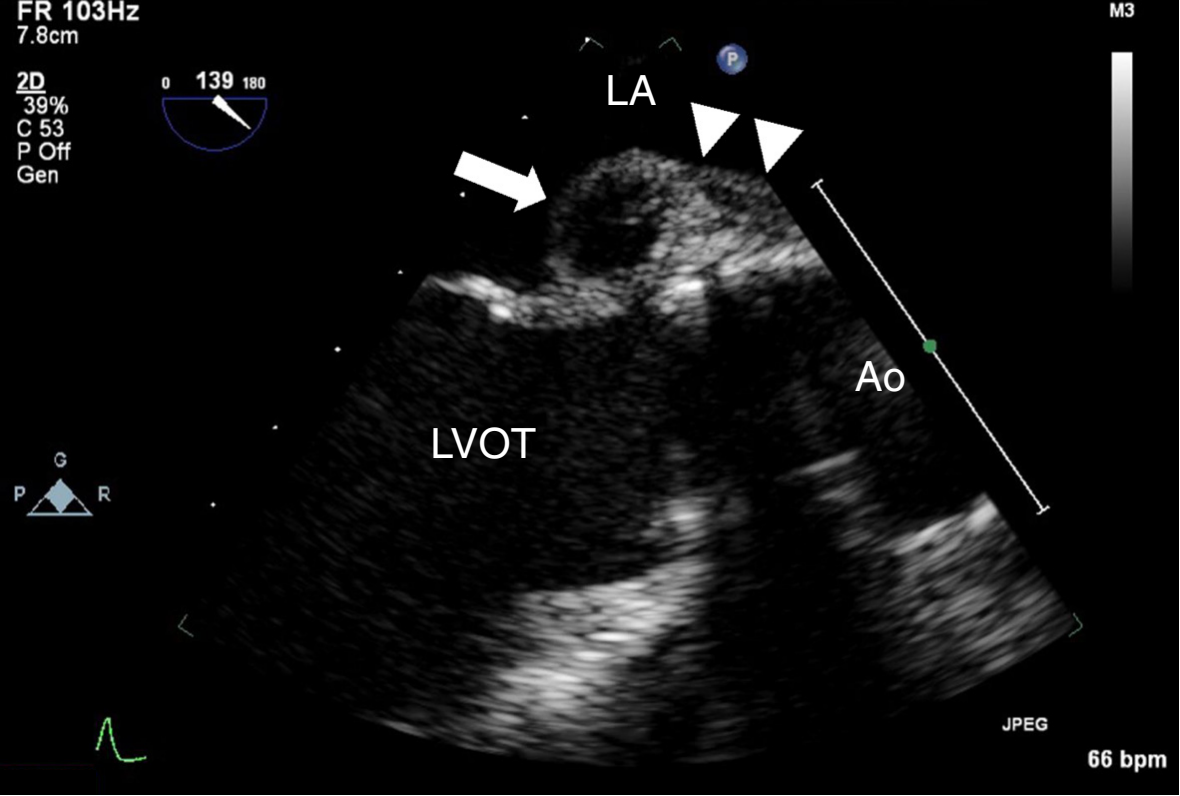
**Transoesophageal Echocardiography illustrating the pseudoaneurysma (arrow) of the left ventricular outflow tract and the thickening of the aortic root (arrowheads).** LA denotes left atrium, LVOT left ventricular outflow tract, Ao Aorta.

A 29-year-old man underwent aortic valve replacement (ATS 29 mm, ATS Medical Inc., Minneapolis, MN, USA) and composite graft implantation for moderate-to-severe aortic valve regurgitation and ectasia of the ascending aorta complicating a congenital bicuspid aortic valve. Eight months later, he presented with a three month history of fatigue, chills, night sweats and myalgias. On admission, he was afebrile with no systemic manifestations of infection. No cutaneous lesions or heart murmur was noted on physical examination. Laboratory analyses revealed mild anaemia with a haemoglobin of 130 g/L (range 140–160 g/l). Platelet and peripheral leukocyte counts were normal as were liver and renal function tests. C-reactive protein level was slightly elevated at 32.4 mg/L. A new first-degree AV block was detected on ECG. Transeosophageal echocardiography demonstrated a thickened wall of the aortic root (11 mm) and a pseudoaneurysm (13 × 18 mm) of the left ventricular outflow tract, interpreted as abscess formation (Figure 1) supporting a diagnosis of prosthetic valve endocarditis with abscess formation and composite graft infection.

After collection of blood cultures, empiric intravenous therapy with vancomycin 1 g bid, amikacin 1 g qd and rifampin 600 mg bid for prosthetic valve endocarditis was started. Two days later, moxifloxacin 400 mg qd was added to cover culture-negative pathogens. One week after hospital admission serologies for *Brucella* spp., *Chlamydia* spp., *Mycoplasma pneumoniae, Coxiella burnetti, Treponema pallidum* and *Borrelia* spp*.* were negative as were stool- and serum-PCR for *Tropheryma whipplei.*

After twelve days of incubation, *Propionibacterium acnes* grew in all (4/4) anaerobic blood culture bottles*.* Despite the recommendation for abscess evacuation and debridement of the composite graft, the patient decided against a surgical procedure.

In addition to MICs (minimum inhibitory concentration), MBCs (minimal bactericidal concentration) were determined in stationary phase [6] (Table 1). Antibiotic therapy was revised with discontinuation of prior therapy and initiation of ceftriaxone 2 g qd and rifampin 600 mg bid. The patient remained afebrile, the CRP concentration returned to normal range and the first-degree AV block resolved. After 7 weeks of intravenous antibiotic therapy, oral therapy was initiated with levofloxacin and rifampin and given for an additional 6 months. The patient remained asymptomatic after cessation of treatment, works full time and vigorously exercises three hours/week. Two sets of blood cultures collected six weeks after completion of treatment remained negative. Serial echocardiography has shown no disease progression (Additional file 1: Figure S1), the peripheral leukocyte count and the CRP-level remained in normal range > 2 years after completion of antibiotic therapy. To our knowledge, this is the first reported case of prosthetic valve endocarditis (PVE) with abscess formation due to *P. acnes* that was successfully managed without surgical therapy.

**Table 1:** Susceptibility tests with growing (MIC) and stationary phase bacteria (MBC)

	**MIC (mg/l)**	**MBCstat (mg/l)**
Ciprofloxacin	0.25	100
Levofloxacin	0.125	-
Rifampicin	0.002	3.12
Ceftriaxone	0.5	250

MBCstat: minimal bactericidal concentration determined with stationary-phase bacteria.

MIC: minimum inhibitory concentration.

## Discussion

Infective endocarditis (IE) due to anaerobic bacteria and especially *P. acnes* is very rare [1,2]: Scant data on incidence and treatment of these infections have been published. In fact, the evidence for treatment of IE due to *P. acnes* is based on case reports and reviews of the literature: In a Korean analysis of 522 patients with positive blood cultures with *P. acnes*, 18 (3.5%) met criteria for bloodstream infection. Only one patient with a ventricular septal defect presented with infective endocarditis [7]. A recent prospective study of 16 consecutive cases of *P. acnes* prosthetic valve endocarditis indicated that the disease might be acquired during heart surgery or could be related to bacteremia of mucocutaneous origin. Diagnosis was often delayed due to oligosymptomatic manifestation, resulting in symptoms associated with progressive prosthetic valve dysfunction leading to heart failure (62%) at the time of diagnosis. The majoritiy of patients had a favourable course (58.3%) when treated with antibiotics and surgical therapy, all patients who initially received antibiotic treatment alone were later in need of surgical intervention due to relapse. Endocarditis related mortality was 18.7% [3]. Our patient was successfully treated with antibiotics, but without surgery. Several factors might explain, why this approach was successful: First, ceftriaxone MBC_stat_ (250 mg/l) determined in our patient with stationary phase bacteria were higher than published peak concentrations in serum (150 mg/l), possibly explaining, potentially in part, the failure of this agent (Table 1) if not combined with rifampin. Bacteria on foreign-bodies (FBI) do not grow exponentially, adhere to the surface and may produce biofilm. These conditions are far away from the conditions used in the test tube to determine MICs. Therefore, we performed MBCs with 4×10^5^ CFU *P. acnes* under anaerobic condition using phosphate-buffered saline as media [6]. Among agents evaluated in susceptibility testing in our patient, only the expected rifampin peak levels exceeded MBC_stat_, an assay that is predictive for successful antimicrobial therapy for FBI [6,8]. Rifampin has an excellent property to kill non-growing, adherent bacteria, and is active against biofilm-forming bacteria, as also shown for *P. acnes*[5]. Second, bacterial biofilm formation is central in the pathogenesis of infections related to foreign material, and *P. acnes* has been shown to form biofilm both in vitro and in vivo [9]. Biofilm formation of 93 *P. acnes* isolates from either invasive infections or skin of healthy people were analysed in a recent Swedish study. Isolates from deep infections produced biofilm, whereas the skin isolates were poor biofilm-producers, which highlights the importance of biofilm formation in *P. acnes* virulence [10]. Third, long-term treatment is commonly required for implant-associated infection, even when signs, symptoms and laboratory parameters returned to normal range after 2–3 weeks after initiating treatment [11]. Recently published data of an in vitro- and animal infection model [5] shows that daptomycin in combination with rifampin followed by levofloxacin and rifampin might be a reasonable antibiotic combination to treat FBI due to *P. acnes*.

## Conclusion

A patient with *P. acnes* prosthetic valve endocarditis with abscess formation of the left ventricular outflow tract was successfully treated with antibiotics without undergoing surgery. A six month treatment with a rifampin and levofloxacin combination was chosen, based on the excellent activity against stationary-phase and adherent bacteria. The patient is considered to be cured based on absence of clinical signs and symptoms, normal laboratory parameters, negative radiology scans and negative blood cultures, determined at site visits over two years after end of treatment.

Written informed consent was obtained from the patient for publication of this case report and any accompanying images.

## Competing interests

The authors declare that they have no competing interests.

## Authors’ contribution

MK wrote the manuscript, evaluated the case and confirmed consistency of data. BAK performed and analyzed echocardiographic analyses and revised the manuscript. LMB served as consultant for treatment of the patient, and revised the manuscript. AFW initiated the paper, performed the in-vitro test, was treating physician and revised the manuscript. All authors’ read and approved the final manuscript.

## Pre-publication history

The pre-publication history for this paper can be accessed here:

http://www.biomedcentral.com/1471-2334/14/105/prepub

## Supplementary Material

Additional file 1: Figure S1 Transthoracic Echocardiography > 2 years after initial diagnosis illustrating unchanged dimensions of the pseudoaneurysma (arrow) of the left ventricular outflow tract. LA denotes left atrium, LVOT left ventricular outflow tract. Click here for file
